# The association between atopic eczema and lymphopenia: Results from a UK cohort study with replication in US survey data

**DOI:** 10.1111/jdv.18841

**Published:** 2023-01-25

**Authors:** Loes M. Hollestein, Morgan Ya Fang Ye, Ky‐Leigh Ang, Harriet Forbes, Kathryn E. Mansfield, Katrina Abuabara, Liam Smeeth, Sinéad M. Langan

**Affiliations:** ^1^ Department of Dermatology Erasmus University Medical Center Rotterdam The Netherlands; ^2^ Department of Research Netherlands Comprehensive Cancer Organization (IKNL) Utrecht The Netherlands; ^3^ Department of Dermatology University of California San Francisco San Francisco California USA; ^4^ Department of Non‐communicable Disease Epidemiology London School of Hygiene and Tropical Medicine London UK; ^5^ Department of Population Health Sciences University of Bristol Bristol UK; ^6^ Health Data Research UK London UK

## Abstract

**Background:**

Lymphocyte skin homing in atopic eczema (AE) may induce lymphopenia.

**Objective:**

To determine if AE is associated with lymphopenia.

**Methods:**

We used UK primary care electronic health records (Clinical Practice Research Datalink GOLD) for a matched cohort study in adults (18 years+) (1997–2015) with at least one recorded lymphocyte count. We matched people with AE to up to five people without. We used multivariable logistic regression to estimate the association between AE and lymphopenia (two low lymphocyte counts within 3 months) and linear mixed effects regression to estimate the association with absolute lymphocyte counts using all available counts. Cox proportional hazard models were used to investigate the effect of lymphopenia on common infections. We replicated the study using US survey data (National Health and Nutrition Examination Survey [NHANES]).

**Results:**

Among 71,731 adults with AE and 126,349 adults without AE, we found an adjusted odds ratio (OR) for lymphopenia of 1.16 (95% CI: 1.09–1.23); the strength of association increased with increasing eczema severity. When comparing all recorded lymphocyte counts from adults with AE (*n* = 1,497,306) to those of people without AE (*n* = 4,035,870) we saw a lower mean lymphocyte (adjusted mean difference −0.047 × 10^9^/L [95% CI: −0.051 to −0.043]) in those with AE. The difference was larger for men, with increasing age, and with increasing AE severity and was present among people with AE not treated with immunosuppressive drugs. In NHANES (*n* = 22,624), the adjusted OR for lymphopenia in adults with AE was 1.30 (95% CI: 0.80–2.11), and the adjusted mean lymphocyte count difference was −0.03 × 10^9^/L (95% CI: −0.07 to 0.02). Despite having a lower lymphocyte count, adjusting for time with lymphopenia, did not alter risk estimates of infections.

**Conclusion:**

Atopic eczema, including increasing AE severity, is associated with a decreased lymphocyte count, regardless of immunosuppressive drug use. Whether the lower lymphocyte count has wider health implications for people with severe eczema warrants further investigation.

## INTRODUCTION

Atopic eczema (AE) is a chronic inflammatory condition affecting up to 20% of children and 10% of the adult population.^1^ AE has the highest global burden in terms of disability adjusted life years of all skin conditions, yet quantitative markers of disease activity and severity are lacking.^2^

Atopic eczema aetiology involves both skin barrier dysfunction and immune dysregulation, for which lymphocytes play a key role.^1^ The immunology literature describes skin homing of lymphocytes (i.e. movement of white blood cells out of the circulating volume to the skin) as a major feature of AE.^3^, ^4^, ^5^ The degree of skin homing may be sufficient to induce detectable lymphopenia on laboratory testing as is evidenced by case reports of lymphopenia in severe AE.^6^, ^7^ Lymphopenia may be a marker of increased infection risk and it complicates initiation and monitoring of currently available broad immunosuppressive systemic therapies to manage AE.^8^

People with AE are known to have increased rates of cutaneous and non‐cutaneous infections.^9^, ^10^ As AE is common and infections can be associated with serious health consequences including morbidity and mortality, understanding whether at a population level, AE is associated with lymphopenia and whether this might lead to increased risk of infection is important. Therefore, we conducted a cohort study to investigate whether adults with AE were more likely to have lymphopenia when compared to people without AE. We undertook exploratory analyses to determine if lymphopenia might be a mediator of increased infection risks and we then externally replicated our findings by repeating the analyses in a separate cohort.

## METHODS

We conducted a matched cohort study comparing the odds of lymphopenia in people with AE to a matched (age, sex, practice) cohort without AE using UK primary care data from Clinical Practice Research Datalink (CPRD GOLD). We then repeated our analysis using US survey data from the National Health and Nutrition Examination Survey (NHANES). Here, we describe the main CPRD study in CPRD in detail and briefly describe the replication in NHANES (Details can be found in the Appendix S1).

### Setting

We used routinely collected UK primary care electronic health record data from CPRD GOLD (9% of the UK population), and linked hospital admissions data from Hospital Episode Statistics (HES), Office for National Statistics mortality data and Index of Multiple Deprivation (IMD) data based on the individual's postcode.^11^ IMD consists of seven components (i.e. income, employment, education, health, crime, barriers to housing and services and living environment) which are weighted and compiled into a single score of deprivation. CPRD data include diagnoses (coded using Read morbidity codes), prescriptions and referrals to specialists. Approximately 80% of CPRD practices have consented to their records being linked to other data sources. HES data includes all NHS‐funded hospital admissions coded using ICD‐10 (international classification of diseases, 10th revision) codes.

### Study population

Adults (≥18 years) registered with a CPRD practice between 1st April 1997 and 31st March 2015, who were eligible for HES linkage were eligible for inclusion.

### Atopic eczema

We defined AE based on at least three medical record codes including a diagnosis code and at least two AE therapy codes (recorded on separate dates), consistent with a validation study showing a positive predictive value in adults of 82%.^12^ AE diagnostic codes were identified in CPRD (using Read codes) and HES (using ICD‐10 codes recorded in the primary diagnosis field of any episode). AE therapies included AE‐related primary care prescriptions: emollients, topical and oral corticosteroids, tacrolimus and systemic immunosuppressants and phototherapy records from primary (CPRD) or secondary (Office of Population Censuses and Surveys [OPCS] Classification of Interventions and Procedures codes in HES) care. Severity of AE was defined as a time‐updated variable (Appendix S1).

### Matched individuals without AE

For each individual with AE, we randomly matched, without replacement, up to five individuals by age (within 15 years), sex, and general practice in calendar date order. People without AE were required to have at least 1 year of follow‐up in CPRD and no history of AE when matched. Any individuals with a diagnosis of AE were included in the pool of eligible people without AE until the date of their AE diagnosis.

### Exclusions

We excluded individuals without a valid lymphocyte count recorded in the primary care records. We also excluded matched sets if either the person with AE or all matched persons without AE did not have any lymphocyte counts.

### Follow‐up

Follow‐up for people with AE began on the latest of: 1st April 1997 (study start), 18th birthday, date they fulfilled our AE diagnosis algorithm, or 1 year after registration with a CPRD practice. Individuals without AE entered the cohort on the same date as the individual with AE whom they were matched to. Follow‐up ended at the earliest of study end date (31 March 2015), death, no longer registered with practice, or practice no longer contributing to CPRD. We included all those contributing at least 1 day of follow‐up.

### Outcome: blood cell counts

We identified lymphocyte counts from CPRD using established methodology.^13^, ^14^ We also included lymphocyte count values without a Read term for lymphocyte count. If multiple lymphocyte counts were recorded for an individual on the same day, we took the mean value. We only used absolute lymphocyte counts and excluded any relative counts (2 out of 12 million lymphocyte counts). We considered lymphocyte counts between 1 × 10^9^ and 4.8 × 10^9^/L as within normal range and <1 × 10^9^/L as lymphopenia. Total white blood cell count, neutrophil count and platelet count were identified as negative controls, as we hypothesized no associations with AE. As immunosuppressive drug use may influence total white blood cell count, total white blood cell count and neutrophil count were performed within patients without any immunosuppressive drug use.

### Covariates

People with and without AE were matched on 15‐year age category and sex. Other covariates included ethnicity, deprivation (quintiles of 2015 IMD), smoking, comorbidities and immunosuppressive drug use, which were taken into account in a relevant time window (Appendix S1).The effect of all covariates on the outcome was assessed statistically. All covariates that influenced the effect estimate by 10% or more were included in the final model. The final models included smoking (lymphopenia and absolute lymphocyte count model) and oral glucocorticoid use (lymphopenia model only) in addition to age and sex (matching variables). All codes used to define outcomes, exposures and covariates are available for download (Data S1).

### Statistical analysis

#### Primary outcome: Lymphopenia

In order to avoid misclassification of lymphopenia based on one accidental finding, we defined lymphopenia as having two low lymphocyte counts (<1 × 10^9^/L) within 3 months. We used logistic regression to compare the odds of lymphopenia in people with AE to people without (Figure S1a). The date of the first low lymphocyte count was considered the date of lymphopenia and was used to define the relevant time window for measuring each covariate.

#### Secondary outcome: Absolute lymphocyte count

In order to take all recorded lymphocyte counts from all individuals into account, we applied a linear mixed effects model (LMM) (Figure S1b). Data were clustered within individuals (all lymphocyte counts for each individual) and within matched sets (people with AE matched to individuals without). Due to the large sample size, a random intercept for each individual, or matched set, was not feasible. Therefore, we included a random intercept for General Practitioner (GP) practice and included the other matching variables (age, sex and calendar time) as fixed covariables in the model. To model the correlation between multiple lymphocyte counts per person during follow‐up, we applied a compound symmetry covariance structure (i.e. all lymphocyte counts for the same individual were equally correlated, regardless of time between the lymphocyte counts), which resulted in the best fitting model based on Akaike's Information Criterion (AIC). Models included time‐varying covariates as described above.

#### Secondary analyses

We stratified models on AE severity and immunosuppressive drug use, regardless of statistical interaction, because we hypothesized that the lymphocyte count would decrease with increasing AE severity and that the association would also be present among people with AE who did not use immunosuppressive drugs. As severe AE is likely to be associated with immunosuppressive drug use, we also applied stratification on immunosuppressive drug use within categories of AE severity. We also investigated whether the effect of AE on lymphopenia was modified by age, sex, smoking and ethnicity (see Appendix S1). Based on the *p*‐value for interaction, the logistic regression model for lymphopenia did not require any further stratification, but the LMM for absolute lymphocyte count was also stratified on age and sex.

Having demonstrated that AE was associated with lymphopenia, we undertook a further post hoc cohort study in CPRD GOLD to investigate whether people with AE were more likely to experience common infections (cellulitis, varicella zoster, gastroenteritis, urinary tract infection) compared to a comparator cohort matched on age, sex and primary care practice, and whether lymphopenia mediated the relationship (Appendix S1).

#### External replication

We replicated our analyses in another population‐based setting, using publicly available data from NHANES, a US population‐based survey. NHANES uses a multistage probability design to select a nationally representative sample of the non‐institutionalized, civilian US population.^15^ Details on AE definition, data on blood samples and analyses can be found in the Appendix S1.

## RESULTS

### Primary outcome: Lymphopenia

**FIGURE 1:**
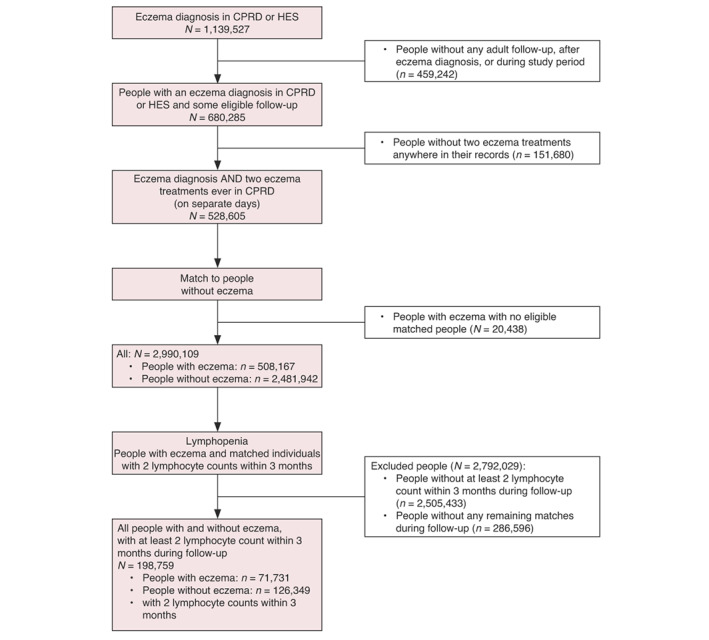
Flow diagram of the lymphopenia analyses (Primary outcome).

In the primary analyses 71,731 adults with AE and 126,349 adults without AE were included (Figure 1, Table 1). Of all people with AE, 4.1% (2909) had lymphopenia compared to 3.7% (4700) without AE and the prevalence of lymphopenia increased with AE severity (Table S1).

**TABLE 1:** Individual characteristics of the study population in the lymphopenia analysis (Primary outcome)

	Adults with atopic eczema		Adults without atopic eczema	
	*N*	%	*N*	%
Total	71,731		126,349	
Follow‐up in years (median, IQR)	9.0 (5.5–13.0)		8.8 (5.4–12.8)	
Age in years (median, IQR)^a^	68 (50–78)		70 (55–79)	
Sex				
Men	24,459	34	44,447	35
Women	47,272	66	81,902	65
Smoking^b^				
No smoker	25,383	35	46,968	37
Current smoker	18,406	26	32,553	26
Ex‐smoker	17,085	24	27,861	22
Missing information	10,857	15	18,967	15
Ethnicity				
White	36,123	50	64,677	51
Other	2859	4	3922	3
Missing information	32,749	46	57,750	46
Socioeconomic deprivation^b^				
1 (low)	17,511	24	30,998	25
2	15,711	22	27,921	22
3	15,147	21	27,432	22
4	12,675	18	22,026	17
5 (high)	10,632	15	17,829	14
Missing information	55	0	143	0
Eczema severity^c^				
Mild	39,269	55		
Moderate	27,829	39		
Severe	4633	6		
Comorbidities				
Asthma^d^	16,549	23	19,033	15
Autoimmune disorders^d^	2564	4	4231	3
Cardiac failure^d^	4667	7	7785	6
Chronic kidney disease^d^	8539	12	15,594	12
Hemopoeitic stem cell transplantation^e^	6	0	11	0
Infections^f^	129	0	191	0
Lymphoproliferative malignancy^e^	190	0	335	0
Sarcoidosis^e^	52	0	11	0
Solid organ cancer^e^	4.070	6	7.748	6
Stress‐related symptoms^c^	288	0	383	0
Immunosuppresive drug use^e^				
Oral glucocorticoids	11,042	15.4	14,514	11.5
Other immunosuppressive drugs	2666	3.7	4097	3.2

*Note*: Numbers indicate the timepoint or window of covariate assessment. The time window refers to the time before the first lymphocyte count + time between first and second lymphocyte count, see Figure S1a. Unless, indicated otherwise, there were no missing values.

^a^ Lymphopenia assessment (second lymphocyte count).

^b^ Cohort entry.

^c^ Time window: 1 year.

^d^ Time window: ever.

^e^ Time window: 2 years.

^f^ Time windows: 3 months for acute infections (influenza) and 2 years for chronic infections (HIV, TBC, viral hepatitis).

**FIGURE 2:**
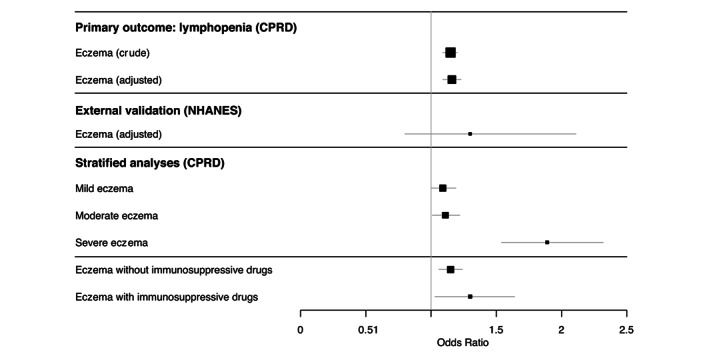
Odds ratios (95% CI) for lymphopenia in people with atopic eczema compared to individuals without atopic eczema (Primary outcome). Larger squares indicate a larger sample size. Lines indicate the 95% confidence intervals. Associated numbers included in the analyses and exact effect estimates, 95% CI and *p*‐values are reported in Tables S2 and S10. All covariates are described in the methods and were assessed in the analyses. Final adjusted models included smoking and oral glucocorticoid use in addition to age and sex (matching variables). External validation in NHANES were adjusted for the same variables as the adjusted model in CPRD. Stratified models were adjusted as well.

The adjusted OR for lymphopenia in people with AE compared to people without AE was 1.16 (95% CI: 1.09–1.23), and increased with increasing AE severity (e.g. OR severe AE: 1.89, 95% CI: 1.54–2.32) (Figure 2, Table S2). Immunosuppressive drugs use was also associated with lymphopenia (OR: 1.15, 95% CI: 1.06–1.24). Patients who had severe eczema, but did not use any immunosuppressive drugs, also had an increased OR for lymphopenia (Table S3). Negative controls (platelet, total white blood cell and neutrophil count) were not associated with AE (Table S4).

### Secondary outcome: Lymphocyte count

The lymphocyte count analyses (LMM) included 1,497,306 lymphocyte counts of 286,906 people with AE and 4,035,870 lymphocyte counts of 866,319 matched individuals without AE (Figure S2, Table S5). The median lymphocyte count of people with AE was 1.80 × 10^9^/L (interquartile range [IQR]: 1.40–2.30) compared to 1.88 × 10^9^/L (IQR: 1.45–2.35) for people without AE.

**FIGURE 3:**
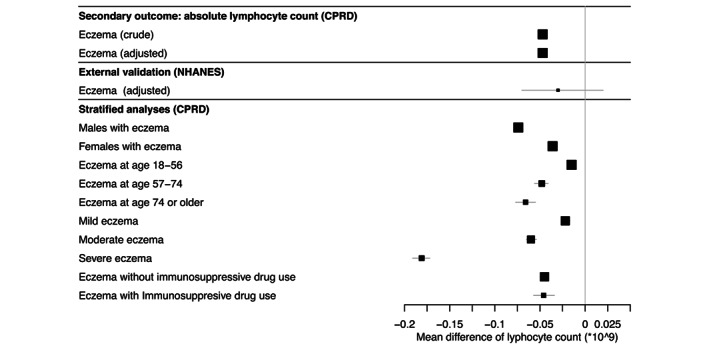
Difference in absolute lymphocyte count (95% CI) in people with atopic eczema patients compared to people without atopic eczema (Secondary outcome). Larger squares indicate a larger sample size. Lines indicate the 95% confidence intervals. Associated numbers included in the analyses and exact effect estimates, 95% CI and *p*‐values are reported in Tables S6 and S10. All covariates are described in the methods and were assessed in the analyses. Final adjusted models included smoking in addition to age and sex (matching variables). External validation in NHANES were adjusted for the same variables as the adjusted model in CPRD. Stratified models were adjusted as well.

Lymphocyte counts of people with AE were lower than lymphocyte counts of people without AE (adjusted mean difference −0.047 × 10^9^/L, 95% CI: 0.051–0.043) (Figure 3, Table S6). The difference was larger for men and older people (Figure 3, Table S6). The lymphocyte count decreased with increasing AE severity. AE regardless of immunosuppressive drug use was associated with decreased lymphocyte count compared to people without AE (Tables S6 and S7).

None of the negative controls (platelet, total white blood cell and neutrophil count) were associated with AE (Table S8).

### Secondary analysis: Infection risks

Using CPRD data (Appendix S2), we estimate the adjusted hazard ratios (HR) comparing rate of common infections in individuals with AE to those without: cellulitis 1.58 (95% CI 1.57–1.60), varicella zoster (VZ) 1.11 (95% CI 1.06–1.16), gastroenteritis 1.33 (95% CI 1.31–1.34), and UTI 1.18 (95% CI 1.17–1.19). HR estimates for all four infections were unchanged after further adjusting for time with lymphopenia.

The absolute excess rate of infection that could be due to AE (attributable risk) was: cellulitis 44.10 per 10,000 person‐years at risk (PYAR) (95% CI 43.25–44.93), VZ 0.65 per 10,000 PYAR (95% CI 0.47–0.82), gastroenteritis 25.39 per 10,000 PYAR (95% CI 24.41–25.87), and UTI 54.73 per 10,000 PYAR (95% CI 54.73–56.75). Sensitivity analyses showed broadly similar effect estimates to those from the main analysis.

### External replication

We included 22,624 participants from NHANES between 1999–2006, in which 5563 participants were part of the 2005–2006 survey wave of which 7%–8% had AE in the past year (Table S9). In the pooled analysis for NHANES 1999–2006, there was a trend towards an inverse association between AE in the past year and lymphocyte count (adjusted mean difference −0.03, 95% CI −0.07, 0.02). There was also a trend towards an increased odds of lymphopenia (adjusted OR 1.30, 95% CI 0.80, 2.11) (Figures 2 and 3, Table S10).

## DISCUSSION

Using data from UK primary care, we have shown that adult AE is associated with both lymphopenia and lower mean lymphocyte counts. We found that the association was larger among individuals with more severe AE, men and older adults, and did not appear to be influenced by immunosuppressive drug use. In a replication study using survey data from the US, we found similar estimates, but with wider confidence intervals that spanned the null. We identified in secondary analyses that adults with AE had increased risks of common infections. In order to address whether or not a reduced lymphocyte count resulted in increased risk of common infections, we adjusted for time with lymphopenia, but this did not result in attenuation of the associations between AE and specific infections.

Most studies investigating blood counts in AE have focused on rates of eosinophilia or anaemia; ours is one of the first to examine lymphopenia in a population‐based setting,^6^, ^16^, ^17^ and it is the largest to examine infections in the UK.^9^, ^10^ Lymphopenia has been recognized in other chronic immune mediated inflammatory disorders, including inflammatory bowel disease (IBD) and rheumatoid arthritis (RA). Like our findings, a study of IBD found that lymphopenia did not explain higher rates of common infections,^18^ though lymphopenia has been associated with both common and severe infections in RA.^19^, ^20^

Strengths of our study include the use of a routinely collected dataset that is representative of the general population of the UK.^21^, ^22^ A validated algorithm^23^ for use in primary care records was used to identify individuals with AE, based on physician diagnosis. A large proportion (97%) of individuals with AE in the UK are treated by their GP,^24^, ^25^ suggesting that most individuals with AE will be identified from their GP records and the probability of selection bias is low. Hence, results of this study are likely to be generalizable to the UK population. We used negative control outcomes comprising other haematological parameters and repeated our lymphopenia analyses in an independent dataset.

The study also has several limitations. Due to the use of routinely collected data, all variables rely on the individual consulting for their condition and their clinician's recording in their health records. Many individuals with lymphopenia may not be tested or have no records in primary care. This is unlikely to differ by exposure status, although surveillance bias may have occurred, as blood tests are likely to vary with immunosuppressive use. This will result in a non‐differential misclassification and an underestimation of time with lymphopenia. An increased rate of infections (indirect cause) may have caused lower lymphocyte counts among AE patients, rather than the AE (direct cause) itself. Although we addressed the temporality of infection and lymphopenia to reduce the possibility of reverse causality we were not able to assess temporality of lymphopenia with AE diagnosis or treatments.

Additionally, time with lymphopenia only accounted for a very small proportion of total follow‐up time, resulting in a lack of power. The infection analyses were powered to detect moderate effect sizes (minimum detectable HR ranging from 1.21 to 2.45 depending on the incidence rate of the infection), however our results were smaller than the minimum detectable HRs. This could explain why the HR and 95% CI of all infections remained the same in the model accounting for potential mediators and the model accounting for lymphopenia. Finally, severity levels were based on therapeutic prescriptions rather than a direct measure of severity, which is a common approach in the dermatologic literature.^26^, ^27^, ^28^, ^29^

Gastroenteritis and UTI are common in the population.^30^, ^31^, ^32^ Individuals may consequently experience mild gastroenteritis or UTI and not report their symptoms to their GP. However, this is unlikely to be differential by AE status and therefore unlikely to affect the hazard ratio. The increased rates of cutaneous and non‐cutaneous infection among individuals with AE could be explained by ascertainment bias. Individuals with AE are more likely to have regular skin checks and report to their GPs for medical attention. Hence, GPs would be more likely to pick up infections among individuals with AE, biasing the HR of infection away from the null.

Our findings may have several important implications for the clinical management and study of AE. A major limitation to population‐based research is the lack of reliable markers of AE disease activity and severity in routinely collected data, and it is possible that data on lymphocyte counts could help to fill this gap. Moreover, lymphocyte counts may be useful to clinicians to monitor disease activity, severity and course. For example, the lymphopenia‐to‐neutrophil ratio has been proposed as a cost‐effective and readily available biomarker to track disease activity in RA and ankylosing spondylitis.^33^ Additionally, clinicians may consider testing prior to commencement of immunosuppressive treatment known to reduce lymphocyte counts to establish baseline values.

Although the period of this study pre‐dates the coronavirus pandemic, it is important to note that lymphopenia has been consistently associated with more severe disease and worse outcomes in COVID‐19.^34^ Although current consensus does not indicate that AE patients are at increased risk of SARS‐CoV‐2 infection or poor outcomes overall,^35^ clinicians may consider checking lymphocyte levels in higher risk subsets of patients. Lymphopenia has been associated with mortality in the general population.^36^ Thus, additional research is needed to understand the long‐term clinical implications in AE.

In summary, we found higher rates of lymphopenia and common infections in adults with AE, though lymphocyte counts were not predictive of increased infection risk. Additional research on the implications of lymphopenia and clinical utility of blood counts is warranted. Knowing that individuals with AE have higher rates of infection can help in the development of a more comprehensive approach to decrease morbidity in individuals with AE and may help guide more targeted vaccination and/or treatment strategies in the future.

## FUNDING INFORMATION

SML was supported by a Wellcome Trust Senior Research Fellowship in Clinical Science (205039/Z/16/Z). The findings and conclusions in this report are those of the authors and do not necessarily represent the views of the funders. SML was also supported by Health Data Research UK (Grant number: LOND1), which is funded by the UK Medical Research Council, Engineering and Physical Sciences Research Council, Economic and Social Research Council, Department of Health and Social Care (England), Chief Scientist Office of the Scottish Government Health and Social Care Directorates, Health and Social Care Research and Development Division (Welsh Government), Public Health Agency (Northern Ireland), British Heart Foundation and Wellcome Trust. SML is an investigator on the European Union Horizon 2020‐funded BIOMAP Consortium (http://www.biomap‐imi.eu/). Funders had no role in the study design, collection, analysis and interpretation of data; in the writing of the report; and in the decision and submitting the article for publication. This research was funded in whole or in part by the Wellcome Trust (G205039/Z/16/Z). For the purpose of Open Access, the author has applied a CC by public copyright licence to any Author Accepted Manuscript (AAM) version arising from this submission.

## CONFLICT OF INTEREST

KA is a consultant for Target RWE.

## Supporting information


Figure S1

Figure S2

Table S1

Table S2

Table S3

Table S4

Table S5

Table S6

Table S7

Table S8

Table S9

Table S10

Appendix S1

Appendix S2

Data S1

## Data Availability

The data that support the findings of this study are available from Clinical Practice Research Datalink (CPRD) and National Health and Nutrition Examination Survey (NHANES). Restrictions apply to the availability of these data, which were used under licence for this study. Data are available from CPRD and NHANES on a licensed basis.
