# Supplementary material for: The association between atopic eczema and lymphopenia: Results from a UK cohort study with replication in US survey data
Source: J Eur Acad Dermatol Venereol. 2023 Jan 25;37(6):1190–8. doi: 10.1111/jdv.18841 (PMC10947025; doi:10.1111/jdv.18841)

Supplementary Figure 1a: Diagram of the method of analysis – Logistic Regression

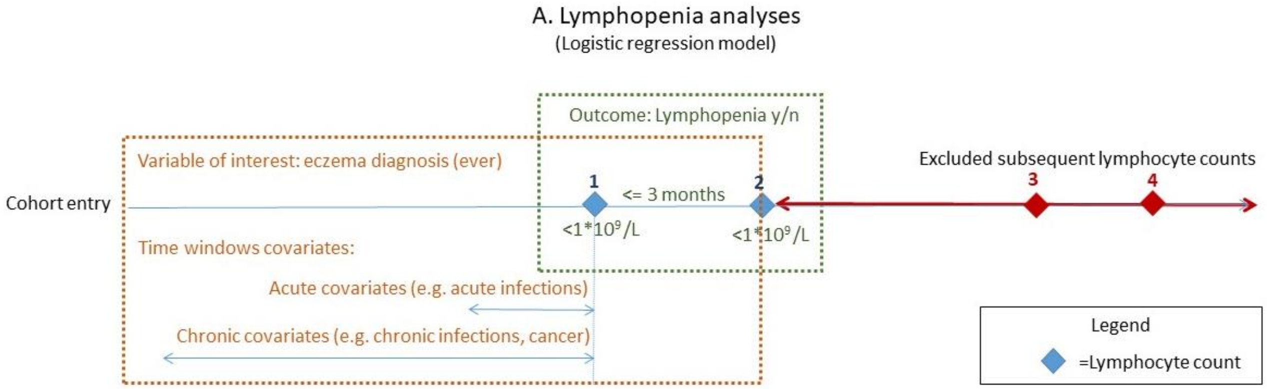

Supplementary Figure 1b: Diagram of the method of analysis – Linear Mixed Model

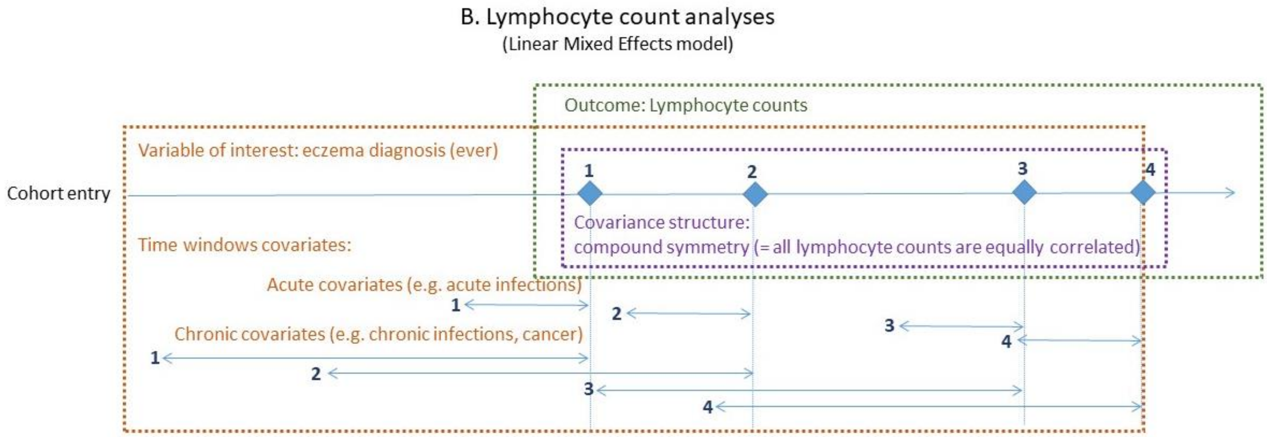

Supplement: Supplementary file 1 — Figure S1 [file JDV-37-1190-s008.pdf]
