# Supplementary material for: The association between atopic eczema and lymphopenia: Results from a UK cohort study with replication in US survey data
Source: J Eur Acad Dermatol Venereol. 2023 Jan 25;37(6):1190–8. doi: 10.1111/jdv.18841 (PMC10947025; doi:10.1111/jdv.18841)
Supplement: Supplementary file 2 — Figure S2 [file JDV-37-1190-s013.docx]

**Supplementary Figure 2:** Flow diagram of the lymphocyte count analyses (secondary outcome)

Eczema diagnosis in CPRD or HES

N=1,139,527

People with an eczema diagnosis in CPRD or HES and some eligible follow-up

N= 680,285

People without any adult follow-up, after eczema diagnosis, or during study period (n=459,242)

Eczema diagnosis AND two eczema treatments ever in CPRD (on separate days)

N= 528,605

People without two eczema treatments anywhere in their records (n=151,680)

Match to people without eczema

All: N=2,990,109

People with eczema: n=508,167

People without eczema: n=2,481,942

People with eczema with no eligible matched individuals (N=20,438)

Lymphocyte count

People with eczema and matched people with at least 1 lymphocyte count

Excluded people (N=1,836,884):

-People without any lymphocyte count during follow-up (n=1,459,126)

-People without any remaining matches during follow-up

(n=377,758)

All people with and without eczema

with at least 1 lymphocyte count during follow-up

N=1,153,225

People with eczema: n=286,906

People without eczema: n=866,319
