# Supplementary material for: The association between atopic eczema and lymphopenia: Results from a UK cohort study with replication in US survey data
Source: J Eur Acad Dermatol Venereol. 2023 Jan 25;37(6):1190–8. doi: 10.1111/jdv.18841 (PMC10947025; doi:10.1111/jdv.18841)
Supplement: Supplementary file 3 — Table S1 [file JDV-37-1190-s010.docx]

**Supplementary Table 1:** Blood cell counts of patients in the lymphopenia analyses

|  | Eczema |  | Non-Eczema |  |
| --- | --- | --- | --- | --- |
|  | N | % | N | % |
| Total | 71,731 |  | 126,349 |  |
|  |  |  |  |  |
| **Lymphopenia** | 2,909 | 4.1% | 4,700 | 3.7% |
| *Lymphopenia according to eczema severity:* |  |  |  |  |
| Mild | 1,402 | 3.6% |  |  |
| Moderate | 1,205 | 4.3% |  |  |
| Severe | 302 | 6.5% |  |  |
| **Other Bloodcounts** |  |  |  |  |
| Two platelet counts < 3 months | 70,332 | 98% | 122,990 | 97% |
| of which both low | 2,398 | 3.3% | 4,409 | 3.5% |
| Two total WBC counts < 3 months | 70,326 | 98% | 123,040 | 97% |
| of which both low | 2,425 | 3.4% | 4,346 | 3.4% |
| Two neutrophil counts < 3 months | 70,448 | 98% | 123,223 | 98% |
| of which both low | 1,441 | 2.0% | 2,776 | 2.2% |
