# Supplementary material for: The association between atopic eczema and lymphopenia: Results from a UK cohort study with replication in US survey data
Source: J Eur Acad Dermatol Venereol. 2023 Jan 25;37(6):1190–8. doi: 10.1111/jdv.18841 (PMC10947025; doi:10.1111/jdv.18841)
Supplement: Supplementary file 4 — Table S2 [file JDV-37-1190-s005.docx]

**Supplementary Table 2**: Logistic regression with lymphopenia as outcome and eczema as exposure

| Models^1^ | Total | Eczema |  | No Eczema |  | OR  Eczema vs no eczema | 95% CI |  | p-value |
| --- | --- | --- | --- | --- | --- | --- | --- | --- | --- |
|  |  | No Lymphopenia | Lymphopenia | No Lymphopenia | Lymphopenia |  |  |  |  |
| **Primary analysis** |  |  |  |  |  |  |  |  |  |
| Crude | 198080 | 68822 | 2909 | 121649 | 4700 | 1.15 | 1.09 | 1.20 | <0.0001 |
| Adjusted^2^ | 168256 | 58710 | 2164 | 103855 | 3527 | 1.16 | 1.09 | 1.23 | <0.0001 |
|  |  |  |  |  |  |  |  |  |  |
| **Stratified adjusted models^3^** |  |  |  |  |  |  |  |  |  |
| **Severity** | 168256 |  |  |  |  |  |  |  |  |
| No Eczema |  |  |  | 103855 | 3527 | 1.00 |  |  |  |
| Mild eczema |  | 32585 | 1039 |  |  | 1.09 | 1.00 | 1.19 | 0.0454 |
| Moderate eczema |  | 22288 | 866 |  |  | 1.11 | 1.01 | 1.22 | 0.0242 |
| Severe eczema |  | 3837 | 259 |  |  | 1.89 | 1.54 | 2.32 | <0.0001 |
|  |  |  |  |  |  |  |  |  |  |
| **Immunosuppressive drugs^4^** | 168256 |  |  |  |  |  |  |  |  |
| No immunosuppressive drugs |  | 48933 | 1537 | 90818 | 2687 | 1.15 | 1.06 | 1.24 | 0.0003 |
| Immunosuppressive drugs |  | 9777 | 627 | 13037 | 840 | 1.30 | 1.03 | 1.64 | 0.0285 |
|  |  |  |  |  |  |  |  |  |  |

^1^ Timepoints and windows for each covariate can be observed from table 1.

^2^ All covariates are described in the methods and were assessed in the analyses. Final adjusted models included smoking and oral glucocorticoid use in addition to age and sex (matching variables).

^3^ The following variables were tested for effect modification: Gender, Age, Smoking, Ethnicity, but none of the p-values for interaction were statistically significant.

^4^ Including both oral glucocorticoids and other immunosuppressive drugs
