# Supplementary material for: The association between atopic eczema and lymphopenia: Results from a UK cohort study with replication in US survey data
Source: J Eur Acad Dermatol Venereol. 2023 Jan 25;37(6):1190–8. doi: 10.1111/jdv.18841 (PMC10947025; doi:10.1111/jdv.18841)
Supplement: Supplementary file 5 — Table S3 [file JDV-37-1190-s015.docx]

**Supplementary Table 3:** Sensitivity analysis of logistic regression with lymphopenia as outcome and atopic eczema as exposure

| Models |  | Eczema |  | No Eczema |  | OR Eczema vs no eczema | 95% CI |  | p-value |
| --- | --- | --- | --- | --- | --- | --- | --- | --- | --- |
|  | Total | Lymphopenia | No Lymphopenia | Lymphopenia | No Lymphopenia |  |  |  |  |
| Stratified adjusted^1^ models |  |  |  |  |  |  |  |  |  |
| **Eczema severity and any immunosuppressive drugs** |  |  |  |  |  |  |  |  |  |
| No eczema, no immunosuppressive drugs | 164729 |  |  | 90818 | 2687 | 1.00 |  |  |  |
| No eczema, any immunosuppressive drugs |  |  |  | 13037 | 840 | 1.85 | 1.66 | 2.07 | <.0001 |
| Mild eczema, no immunosuppressive drugs |  | 28502 | 840 |  |  | 1.15 | 1.04 | 1.27 | 0.0044 |
| Mild eczema, any immunosuppressive drugs |  | 4083 | 199 |  |  | 1.76 | 1.43 | 2.16 | <.0001 |
| Moderate eczema, no immunosuppressive drugs |  | 19091 | 702 |  |  | 1.20 | 1.08 | 1.33 | 0.0008 |
| Moderate eczema, any immunosuppressive drugs |  | 3197 | 164 |  |  | 1.62 | 1.29 | 2.03 | <.0001 |
| Severe eczema, no immunosuppressive drugs |  | 1546 | 46 |  |  | 1.27 | 0.84 | 1.93 | 0.2505 |
| Severe eczema, any immunosuppressive drugs |  | 2291 | 213 |  |  | 3.06 | 2.41 | 3.89 | <.0001 |
|  |  |  |  |  |  |  |  |  |  |

^1^ Models were adjusted for the same confounders as the main analysis for lymphopenia: matched on age and sex and adjusted for smoking and oral glucocorticoid use
