# Supplementary material for: The association between atopic eczema and lymphopenia: Results from a UK cohort study with replication in US survey data
Source: J Eur Acad Dermatol Venereol. 2023 Jan 25;37(6):1190–8. doi: 10.1111/jdv.18841 (PMC10947025; doi:10.1111/jdv.18841)
Supplement: Supplementary file 6 — Table S4 [file JDV-37-1190-s003.docx]

**Supplementary Table 4 :** Negative controls of logistic regression with lymphopenia as outcome and eczema as exposure

| Models |  | Eczema |  | No Eczema |  | OR event vs no event | 95% CI |  | p-value |
| --- | --- | --- | --- | --- | --- | --- | --- | --- | --- |
|  | Total | Event^1^ | No Event | Event^1^ | No Event |  |  |  |  |
| Adjusted^2^ model, platelets | 146865 | 54843 | 1653 | 87597 | 2772 | 0.96 | 0.90 | 1.03 | 0.2330 |
| Adjusted^2^ model, total white blood cell count | 146828 | 54423 | 2053 | 86967 | 3385 | 1.00 | 0.94 | 1.06 | 0.9317 |
|  |  |  |  |  |  |  |  |  |  |
| Only among patients without any oral glucocorticoid or any other immunosuppressant drug use |  |  |  |  |  |  |  |  |  |
| Adjusted model^2^, total white blood cell count | 109777 | 42099 | 1657 | 63515 | 2506 | 0.98 | 0.92 | 1.05 | 0.6244 |
| Adjusted model^2^, neutrophil count | 109582 | 42636 | 1047 | 64144 | 1755 | 0.87 | 0.81 | 0.94 | 0.0007 |
|  |  |  |  |  |  |  |  |  |  |

^1^ An event was defined in the same way as lymphopenia: 2 low counts within 3 months. The following thresholds were used: <150 *10^9^ cells/L for platelets, <4 *10^9^ cells/L for total white blood cell count, and <2 *10^9^ cells/L for neutrophil count.

^2^ Models were adjusted for the same confounders as the main analysis for lymphopenia: matched on age and sex and adjusted for smoking and oral glucocorticoid use. Models excluding patients with oral glucocorticoid use were not adjusted for oral glucocorticoid use.
