# Supplementary material for: The association between atopic eczema and lymphopenia: Results from a UK cohort study with replication in US survey data
Source: J Eur Acad Dermatol Venereol. 2023 Jan 25;37(6):1190–8. doi: 10.1111/jdv.18841 (PMC10947025; doi:10.1111/jdv.18841)
Supplement: Supplementary file 7 — Table S5 [file JDV-37-1190-s001.docx]

**Supplementary Table 5:** Patient characteristics of the lymphocyte count analysis

|  | Eczema |  | Non-Eczema |  |
| --- | --- | --- | --- | --- |
|  | N | % | N | % |
| Total number of patients | In |  | 866,319 |  |
|  |  |  |  |  |
| Follow-up | 7.2  (4.0-11.6) |  | 7.3  (4.1-11.4) |  |
| Age^2^ | 54  (38-70) |  | 58  (42-71) |  |
| Sex |  |  |  |  |
| Men | 101,904 | 36% | 299,259 | 35% |
| Women | 185,002 | 64% | 567,060 | 65% |
| Smoking^1^ |  |  |  |  |
| No smoker | 111,780 | 39% | 352,637 | 41% |
| Current smoker | 77,632 | 27% | 235,348 | 27% |
| Ex-smoker | 69,315 | 24% | 194,979 | 23% |
| Missing information | 28,179 | 10% | 83,355 | 10% |
| Ethnicity |  |  |  |  |
| Caucasian | 133,343 | 46% | 414,871 | 48% |
| Other | 15,267 | 5% | 37,606 | 4% |
| Missing information | 138,296 | 48% | 413,842 | 48% |
| Socioeconomic status^1^ |  |  |  |  |
| 1 (low) | 69,722 | 24% | 212,474 | 25% |
| 2 | 62,785 | 22% | 192,729 | 22% |
| 3 | 58,830 | 21% | 180,764 | 21% |
| 4 | 50,969 | 18% | 150,796 | 17% |
| 5 (high) | 44,317 | 15% | 128,655 | 15% |
| Missing information | 283 | 0% | 901 | 0% |
| **Lymphocyte counts during follow-up** |  |  |  |  |
| Frequency |  |  |  |  |
| 1 | 72,791 | 25% | 239,415 | 28% |
| 2-4 | 130,080 | 45% | 405,732 | 47% |
| 5-10 | 52,090 | 18% | 145,117 | 17% |
| >10 | 31,945 | 11% | 76,055 | 9% |
| **Other Blood counts** |  |  |  |  |
| At least one platelet count | 272,586 | 95% | 812,230 | 94% |
| Platelet count (median, IQR) | 257  (214-309) |  | 255  (212-305) |  |
| At least one total WBC count | 272,457 | 95% | 811,446 | 94% |
| Total WBC count (median, IQR) | 6.90  (5.69-8.50) |  | 6.90  (5.63-8.46) |  |
| At least one neutrophil count | 272,668 | 95% | 812,841 | 94% |
| Neutrophil count (median, IQR) | 4.10  (3.20-5.40) |  | 4.10  (3.15-5.38) |  |
| Total number of lymphocyte counts | 1,497,306 |  | 4,035,870 |  |
| Lymphocyte count (*10^6^, median, IQR) | 1.80 (1.40-2.30) |  | 1.88 (1.45-2.35) |  |
| Density of lymphocyte count in the preceding year (median, IQR) | 1 (0-2) |  | 1 (0-2) |  |
| **Eczema Severity^3^** |  |  |  |  |
| Mild | 741,665 | 50% |  |  |
| Moderate | 542,807 | 36% |  |  |
| Severe | 212,834 | 14% |  |  |
| **Comorbidities associated with Lymphopenia^7^** |  |  |  |  |
| Autoimmune disorders^5^ | 126,376 | 8.4% | 312,065 | 7.7% |
| Cardiac failure^5^ | 85,269 | 5.7% | 207,519 | 5.1% |
| Chronic Kidney Disease^5^ | 186,605 | 12% | 506,699 | 13% |
| Hemopoeitic Stem Cell Transplantation^4^ | 152 | 0.0% | 452 | 0.0% |
| Infections^6^ | 2,072 | 0.1% | 4,648 | 0.1% |
| Lymphoproliferative Malignancy^4^ | 3,895 | 0.3% | 9,091 | 0.2% |
| Sarcoidosis^4^ | 1,251 | 0.1% | 2,709 | 0.1% |
| Solid organ cancer^4^ | 65,691 | 4.4% | 180,479 | 4.5% |
| Stress-related symptoms^3^ | 4,912 | 0.3% | 11,337 | 0.3% |
| **Other Comorbidities** |  |  |  |  |
| Asthma^5^ | 363,419 | 24% | 627,514 | 16% |
| **Immunosuppresive drug use^4^** |  |  |  |  |
| Oral glucocorticoids | 235,347 | 16% | 476,417 | 12% |
| Other immunosuppressive drugs | 157,153 | 10% | 360,771 | 9% |

Numbers indicate the timepoint or window of covariate assessment. The covariates are taken into account in a time-varying way at the date of each lymphocyte count in the analysis (supplementary figure 3), but presented here over the whole follow-up time. The number corresponds to the time window before each lymphocyte count used in the main analysis.

^1^ Cohort entry

^2^ At the date of the lymphocyte count, value at the first date presented in this table

^3^ Time window: 1 year

^4^ Time window: 2 years

^5^ Time window: ever

^6^ Time window: 3 months for acute infections and 2 years for chronic infections.

^7^ Percentage is shown as percentage of total lymphocyte counts instead of percentage of patients, because patients can have multiple lymphocyte counts for which comorbidities were taken into account in a time-varying way.
