# Supplementary material for: The association between atopic eczema and lymphopenia: Results from a UK cohort study with replication in US survey data
Source: J Eur Acad Dermatol Venereol. 2023 Jan 25;37(6):1190–8. doi: 10.1111/jdv.18841 (PMC10947025; doi:10.1111/jdv.18841)
Supplement: Supplementary file 8 — Table S6 [file JDV-37-1190-s011.docx]

**Supplementary Table 6:** Linear Mixed Model with each absolute lymphocyte count during follow-up as outcome and eczema as exposure

| Models^1,2^ | Eczema |  |  | No Eczema |  |  | Beta Eczema vs No eczema | 95% CI |  | p-value |
| --- | --- | --- | --- | --- | --- | --- | --- | --- | --- | --- |
|  | N | Unadjusted mean (*10^9^/L) | SD  (*10^9^/L) | N | Unadjusted mean  (*10^9^/L) | SD  (*10^9^/L) | (*10^9^/L) | (*10^9^/L) | (*10^9^/L) |  |
| **Primary analysis** |  |  |  |  |  |  |  |  |  |  |
| Crude | 1,497,306 | 1.956 | 1.183 | 4,035,870 | 2.002 | 1.285 | -0.047 | -0.051 | -0.043 | <.0001 |
| Adjusted^3^ | 1,315,233 | 1.975 | 1.165 | 3,555,672 | 2.017 | 1.253 | -0.047 | -0.051 | -0.043 | <.0001 |
|  |  |  |  |  |  |  |  |  |  |  |
| **Stratified adjusted models^4^** |  |  |  |  |  |  |  |  |  |  |
| **Gender** |  |  |  |  |  |  |  |  |  |  |
| Male | 411,780 | 1.941 | 1.332 | 1,112,848 | 2.005 | 1.482 | -0,074 | -0,083 | -0,066 | <.0001 |
| Female | 903,453 | 1.991 | 1.080 | 2,442,824 | 2.022 | 1.134 | -0,036 | -0,041 | -0,032 | <.0001 |
| **Age** |  |  |  |  |  |  |  |  |  |  |
| 18-56 | 566,309 | 2.057 | 0.898 | 1,332,732 | 2.067 | 0.902 | -0.015 | -0.019 | -0.011 | <.0001 |
| 57-73 | 392,485 | 1.997 | 1.222 | 1,173,685 | 2.064 | 1.294 | -0.048 | -0.056 | -0.041 | <.0001 |
| ≥74 | 356,439 | 1.820 | 1.429 | 1,049,255 | 1.900 | 1.549 | -0.066 | -0.077 | -0.055 | <.0001 |
| **Severity** |  |  |  |  |  |  |  |  |  |  |
| No Eczema |  |  |  | 3,555,672 | 2.017 | 1.253 |  |  |  |  |
| Mild eczema | 655,027 | 2.031 | 1.199 |  |  |  | -0.022 | -0.026 | -0.017 | <.0001 |
| Moderate eczema | 464,940 | 1.983 | 1.171 |  |  |  | -0.060 | -0.065 | -0.054 | <.0001 |
| Severe eczema | 195,266 | 1.768 | 0.999 |  |  |  | -0.181 | -0.191 | -0.172 | <.0001 |
| **Immunosuppressive drug use** |  |  |  |  |  |  |  |  |  |  |
| No immunosuppressive drug use | 1,025,389 | 2.008 | 1.180 | 2,942,041 | 2.047 | 1.266 | -0.045 | -0.050 | -0.041 | <.0001 |
| Immunosuppressive drug use | 289,844 | 1.858 | 1.101 | 613,631 | 1.873 | 1.179 | -0.046 | -0.057 | -0.034 | <.0001 |

^1^ Timepoints and windows for each covariate can be observed from table 1b.

^2^ Matching was taken into account using a random intercept for GP practice and including the other matching variables in the mean model (age, gender, calendar year). Correlation between lymphocyte counts of each patient was taken into account by using a compound symmetry variance-covariance matrix.

^3^ The following variables were tested for confounding (influence of beta >10%): Smoking, social economic status, Autoimmune disorders, Cardiac failure Chronic kidney disease, Hemopoeitic stem cell transplantation, Infections, Inflammatory bowel disease, Lymphoproliferative malignancy, Sarcoidosis, Solid organ cancer, Stress-related symptoms, oral glucocorticoid use, other immunosuppressants. Only smoking was a confounder and included in the adjusted model, in addition to the exposure and matching variables in the mean model.

^4^ The following variables were tested for effect modification: Gender, Age, Smoking, Immunosuppresive drug use. The p-value for interaction was statistically significant for gender, age and immunosuppressive drug use.
