# Supplementary material for: The association between atopic eczema and lymphopenia: Results from a UK cohort study with replication in US survey data
Source: J Eur Acad Dermatol Venereol. 2023 Jan 25;37(6):1190–8. doi: 10.1111/jdv.18841 (PMC10947025; doi:10.1111/jdv.18841)
Supplement: Supplementary file 9 — Table S7 [file JDV-37-1190-s009.docx]

**Supplementary Table 7:** Sensitivity analysis of the Linear Mixed Model with each absolute lymphocyte count during follow-up as outcome and eczema as exposure

| **Stratified adjusted^1^ models** | | Eczema |  |  | No Eczema |  |  | Beta Eczema vs No eczema | 95% CI |  | p-value |
| --- | --- | --- | --- | --- | --- | --- | --- | --- | --- | --- | --- |
|  | | N | Unadjusted mean (*10^9^/L) | SD  (*10^9^/L) | N | Unadjusted mean  (*10^9^/L) | SD  (*10^9^/L) | (*10^9^/L) | (*10^9^/L) | (*10^9^/L) |  |
| **Eczema severity and drug use combined** | |  |  |  |  |  |  |  |  |  |  |
| Eczema | Current use of immunosuppressive drug |  |  |  |  |  |  |  |  |  |  |
| No | No |  |  |  | 2,942,041 | 2.047 | 1.266 |  |  |  |  |
| No | Yes | 582,439 | 2.029 | 1.176 | 613,631 | 1,873 | 1,179 | -0.051 | -0.055 | -0.048 | <.0001 |
| Mild | No | 72,588 | 2.053 | 1.368 |  |  |  | -0.029 | -0.033 | -0.024 | <.0001 |
| Mild | Yes | 400,144 | 1.984 | 1.167 |  |  |  | -0.013 | -0.022 | -0.005 | 0.0021 |
| Moderate | No | 64,796 | 1.979 | 1.196 |  |  |  | -0.065 | -0.071 | -0.060 | <.0001 |
| Moderate | Yes | 42,806 | 1.959 | 1.341 |  |  |  | -0.070 | -0.079 | -0.061 | <.0001 |
| Severe | No | 152,460 | 1.714 | 0.872 |  |  |  | -0.115 | -0.129 | -0.102 | <.0001 |
| Severe | Yes | 582,439 | 2.029 | 1.176 |  |  |  | -0.218 | -0.228 | -0.207 | <.0001 |
|  | |  |  |  |  |  |  |  |  |  |  |

^1^ Models were adjusted for the same confounders as the main analysis for lymphocyte counts: matched on age and sex and adjusted for smoking
