# Supplementary material for: The association between atopic eczema and lymphopenia: Results from a UK cohort study with replication in US survey data
Source: J Eur Acad Dermatol Venereol. 2023 Jan 25;37(6):1190–8. doi: 10.1111/jdv.18841 (PMC10947025; doi:10.1111/jdv.18841)
Supplement: Supplementary file 10 — Table S8 [file JDV-37-1190-s006.docx]

**Supplementary Table 8:** Negative controls of Linear Mixed Model with each absolute lymphocyte count during follow-up as outcome and eczema as exposure

| Models | Eczema |  |  | No Eczema |  |  | Beta Eczema vs No eczema | 95% CI |  | p-value |
| --- | --- | --- | --- | --- | --- | --- | --- | --- | --- | --- |
|  | N | Unadjusted mean (*10^9^/L) | SD  (*10^9^/L) | N | Unadjusted mean  (*10^9^/L) | SD  (*10^9^/L) | (*10^9^/L) | (*10^9^/L) | (*10^9^/L) |  |
| Adjusted^1^ model, **platelets** | 1,307,845 | 270 | 84 | 3,471,142 | 267 | 84 | 1.1 | 0.8 | 1.4 | <.0001 |
| Adjusted^1^ model, **total white blood cell count** | 1,308,118 | 7.307 | 2.604 | 3,470,820 | 7.285 | 2.623 | 0.023 | 0.014 | 0.032 | <.0001 |
|  |  |  |  |  |  |  |  |  |  |  |
| **Only among patients without any oral glucocorticoid or any other immunosuppressant drug use** | | | | | | | | | | |
| Adjusted model^1^, **total white blood cell** count | 1,020,985 | 7.153 | 2.465 | 2,872,982 | 7.164 | 2.500 | -0.005 | -0.014 | 0.005 | 0.3344 |
| Adjusted model^1^, **neutrophil count** | 982,362 | 4.332 | 1.943 | 2,778,623 | 4.332 | 1.961 | 0.001 | -0.007 | 0.008 | 0.8899 |
|  |  |  |  |  |  |  |  |  |  |  |

^1^ Models were adjusted for the same confounders as the main analysis for lymphocyte counts: matched on age and sex and adjusted for smoking
