# Supplementary material for: The association between atopic eczema and lymphopenia: Results from a UK cohort study with replication in US survey data
Source: J Eur Acad Dermatol Venereol. 2023 Jan 25;37(6):1190–8. doi: 10.1111/jdv.18841 (PMC10947025; doi:10.1111/jdv.18841)
Supplement: Supplementary file 11 — Table S9 [file JDV-37-1190-s002.docx]

**Supplementary Table 9:** NHANES Participant Characteristics

|  | Adults (18+) | |
| --- | --- | --- |
|  | **2005-2006 (N=5,563)** | **1999-2006 (N=22,624)** |
| Age (years), median (IQR) | 42 (27-62) | 44 (28-64) |
| Sex |  |  |
| Male | 2,675 (48.09) | 10,766 (47.59) |
| Female | 2,888 (51.91) | 11,858 (52.41) |
| Poverty-income ratio |  |  |
| Less than 1.3 | 1,505 (27.05) | 6,257 (27.66) |
| 1.3 to less than 3.5 | 2,044 (36.74) | 7,947 (35.13) |
| 3.5 or greater | 1,720 (30.92) | 6,440 (28.47) |
| Missing | 294 (5.28) | 1,980 (8.75) |
| Race/ethnicity |  |  |
| Non-Hispanic White | 2,633 (47.33) | 10,935 (48.33) |
| Non-Hispanic Black | 1,341 (24.11) | 4,799 (21.21) |
| Mexican American | 1,185 (21.3) | 5,198 (22.98) |
| Other | 233 (4.19) | 758 (3.35) |
| Other Hispanic | 171 (3.07) | 934 (4.13) |
| Smoking |  |  |
| Never smoked | 2,625 (47.19) | 10,530 (46.54) |
| Ex-smoker | 1,259 (22.63) | 5,350 (23.65) |
| Current smoker | 1,089 (19.58) | 4,390 (19.4) |
| Missing | 590 (10.61) | 2,354 (10.4) |
| Glucocorticoid use |  |  |
| No | 5,479 (98.49) | 22,243 (98.32) |
| Yes | 84 (1.51) | 381 (1.68) |
| Doctor diagnosed ever having eczema |  |  |
| No | 5,211 (93.67) | - |
| Yes | 344 (6.18) | - |
| Missing | 8 (0.14) | - |
| Eczema in the past year |  |  |
| No | 5,160 (92.76) | 15,824 (69.94) |
| Yes | 400 (7.19) | 1,838 (8.12) |
| Missing | 3 (0.05) | 4,962 (21.93) |
| Flexural eczema in the past year |  |  |
| No | 5,320 (95.63) | - |
| Yes | 240 (4.31) | - |
| Missing | 3 (0.05) | - |
| Lymphopenia |  |  |
| No | 4,932 (88.66) | 19,644 (86.83) |
| Yes | 73 (1.31) | 350 (1.55) |
| Missing | 558 (10.03) | 2,630 (11.62) |
| Low white blood cell count |  |  |
| No | 4,740 (85.21) | 18,880 (83.45) |
| Yes | 287 (5.16) | 1,223 (5.41) |
| Missing | 536 (9.64) | 2,521 (11.14) |
