# Supplementary material for: The association between atopic eczema and lymphopenia: Results from a UK cohort study with replication in US survey data
Source: J Eur Acad Dermatol Venereol. 2023 Jan 25;37(6):1190–8. doi: 10.1111/jdv.18841 (PMC10947025; doi:10.1111/jdv.18841)
Supplement: Supplementary file 12 — Table S10 [file JDV-37-1190-s014.docx]

**Supplementary Table 10.** NHANES Regression results

| **1999-2006^3^** | | | | |
| --- | --- | --- | --- | --- |
| **Eczema in the past 12 months** | | | | |
| Lymphocyte count^1^ | N | mean difference (95%CI) | | |
|  | 14,616 | -0.03 | -0.07 | 0.02 |
| Lymphopenia^2^ | N | OR (95%CI) | | |
|  | 14,616 | 1.30 | 0.80 | 2.11 |
|  |  |  |  |  |
| **2005-2006 only (additional eczema questions available)** | | | | |
| **Eczema in the past 12 months** | | | | |
| Lymphocyte count^1^ | N | mean difference (95%CI) | | |
|  | 4,459 | -0.01 | -0.11 | 0.10 |
| Lymphopenia^2^ | N | OR (95%CI) | | |
|  | 4,459 | 2.43 | 0.87 | 6.81 |
| **Flexural dermatitis in the past 12 months** | | | | |
| Lymphocyte count^1^ | N | mean difference (95%CI) | | |
|  | 4,459 | -0.03 | -0.14 | 0.08 |
| Lymphopenia^2^ | N | OR (95%CI) | | |
|  | 4,459 | 0.53 | 0.22 | 1.24 |
| **Self-report of a physician diagnosis of eczema** | | | | |
| Lymphocyte count^1^ | N | mean difference (95%CI) | | |
|  | 4,454 | -0.02 | -0.09 | 0.06 |
| Lymphopenia^2^ | N | OR (95%CI) | | |
|  | 4,454 | 1.06 | 0.21 | 5.48 |

^1^ Adjusted for sex, age, smoking

^2^ Adjusted for sex, age, smoking, and glucocorticoids

^3^ Additionally adjusted for survey wave
