# Supplementary material for: The association between atopic eczema and lymphopenia: Results from a UK cohort study with replication in US survey data
Source: J Eur Acad Dermatol Venereol. 2023 Jan 25;37(6):1190–8. doi: 10.1111/jdv.18841 (PMC10947025; doi:10.1111/jdv.18841)
Supplement: Supplementary file 13 — Appendix S1 [file JDV-37-1190-s012.docx]

Supplementary Methods

# **CPRD Supplementary methods main analyses**

## CPRD Severity of atopic eczema

Severity of atopic eczema was defined as a time-updated variable for individuals with atopic eczema. Individuals with atopic eczema were considered to have mild eczema by default. They were classified as having moderate atopic eczema at the first of: a second potent topical corticosteroid treatment within one year or a first calcineurin inhibitor treatment. Individuals were classified as having severe atopic eczema at the first of: a systemic immunosuppressant treatment; a phototherapy code in CPRD or Hospital Episode Statistics; or a referral for atopic eczema. Once defined as moderate, people with atopic eczema remained as such unless they developed severe atopic eczema; once defined as severe, people with atopic eczema remained as such, similar to established approaches for defining severity in psoriasis studies ^1^.At any given point during follow-up, people with atopic eczema therefore belonged to one of three severity categories: mild, moderate, or severe.

## Covariates:

#### Smoking and ethnicity

Smoking was defined by using the medical codes from CPRD. Ethnicity was not available for a large proportion of all subjects. Therefore, we repeated the main analyses in a subset with known ethnicity and assessed the influence of ethnicity on the odds ratio of lymphopenia in a subset. We observed that the effect estimate did not change with >10% and thus ethnicity was not included in our final model.

#### Age

As people without AE were not exactly matched on age, but within 15-year age categories, we also included age in the models as a continuous variable.

#### Comorbidities and Immunosuppresive drugs

For all comorbidities (e.g., autoimmune disorders, asthma, chronic diseases, systemic infections, lymphoproliferative malignancies and solid organ cancer) and immunosuppressive drug use (e.g., oral glucocorticoids, chemotherapy, biologicals, immunotherapy), a relevant time window was defined *a priori* based on the likelihood of association with a decreased lymphocyte count: three months for acute infections, 1 year for stress-related symptoms (e.g. acute post-traumatic stress state, crisis state); two years for chronic infections (Human Immunodeficiency Virus [HIV], Tuberculose [TBC], viral hepatitis), haemopoietic stem cell transplantation, lymphoproliferative malignancy, sarcoidosis, solid organ cancers and immunosuppressive drug use; and ever for chronic disorders, such as autoimmune disorders, cardiac failure and inflammatory bowel disease (Supplementary Figure 1a and 1b of the main manuscript).

# **Secondary analysis focused on infection risk (CPRD)**

We conducted a cohort study using routinely collected primary care (Clinical Research Practice Datalink [CPRD] GOLD electronic health record data to investigate whether people with AE were more likely to experience common infections (cellulitis, varicella zoster, gastroenteritis, urinary tract infection) compared to a comparator cohort matched on age, sex, and primary care practice, and whether lymphopenia mediated the relationship.

## Study population

We identified adults (≥18 years) with atopic eczema (AE) and a matched cohort (age, sex, general practice) of individuals without eczema. To be eligible for inclusion individuals had to be at least 18 years of age and registered with a CPRD GOLD practice between 2^nd^ January 1997 and 31^st^ December 2019. We individually matched people with AE to a random selection of up to five individuals with no prior record of a diagnostic morbidity code for AE, without replacement, on age, sex, and primary care practice in calendar date order. Individuals in the matched cohort entered the study on the same day as their matched individual with AE.

We excluded individuals with a previous record for cellulitis and varicella zoster in the primary care records from analyses with the respective infection as the outcome. Participants with previous cellulitis or varicella zoster code before entry into the study were excluded to minimize information bias. However, individuals with previous gastroenteritis or UTI before entry into study were included as we expect many participants would have had previous gastroenteritis or UTI as these are common recurring events, which could lead to selection bias if those individuals were excluded.

All analyses included valid matched sets only (i.e., Matched set without an individual with AE or no matched individuals without AE were also excluded). Individuals with AE were entered in the cohort at the latest of (Supplementary Methods Figure 1): 1) 18^th^ birthday; 2) Study start (2^nd^ January 1997); 3) One year after registration with a CPRD practice meeting CPRD quality control standards; and the date they met in the AE algorithm. Individuals without AE entered the study on the same day as their matched individual with AE.

**Supplementary Methods Figure 1: Study Design**

Visual representation of cohort entry, where the cohort entry date is selected after application of exclusion criteria, and follow-up process


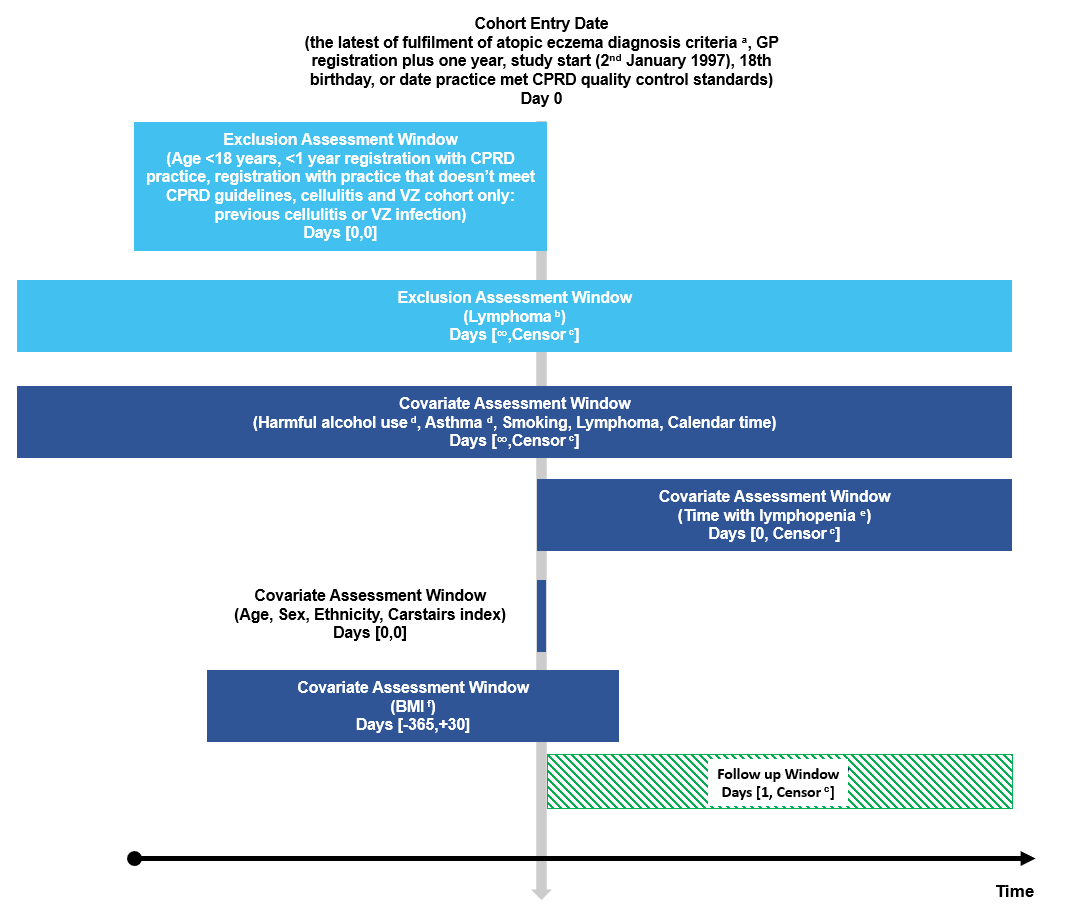


1. AE diagnosis criteria are based on a validated algorithm [33]. Unexposed individuals are matched on age, sex, date of entry into cohort and GP practice.
2. Individuals with a diagnosis of lymphoma were excluded in the secondary analysis
3. Individuals will exit the study at the earliest of 1) death; 2) date they are no longer with their GP practice; 3) date GP practice stops contributing to CPRD; 4) Study end date (31^st^ December 2019); 5) specific infection diagnosis (cellulitis, varicella zoster, gastroenteritis, or urinary tract infection); or 6) Unexposed cohort only: date individual had a record of an AE diagnostic code
4. Asthma and harmful alcohol use are time-updated variables, with status changing on the individuals first asthma or harmful alcohol use.
5. Time with lymphopenia was defined as 183.63 days after a test result indicating lymphopenia. If second blood test demonstrating lymphopenia was recorded within the 6 months, individuals were considered to have lymphopenia for a further 183.63 days after time of second blood test (and so on for subsequent test).
6. Identified using an algorithm where records identified within -1 year to +1 month of the index date are regarded as the best, +1 month to +1 year from the index date as second best, the nearest prior to the year before the index date as the third best, and within +1 year from the index date as the worst.

AE = Atopic eczema; BMI = Body mass index; CPRD = Clinical Practice Research Datalink; GP = General Practice; VZ = varicella zoster

## Atopic eczema

We used an existing algorithm, ^2^ with a positive predictive value of 82%, to identify individuals with AE. Individuals were identified as having AE if they had a record of at least one diagnostic morbidity code for AE and at least two records for AE therapies (including prescriptions for topical agents and systemic treatments) recorded on separate days. AE therapies included prescriptions for emollients, topical and oral corticosteroids, tacrolimus and systemic immunosuppressants (methotrexate, ciclosporin, mycophenolate mofetil, azathioprine), and phototherapy.

## Outcomes

Our outcomes were specific common cutaneous and non-cutaneous infections that are frequently managed by and can be reliably diagnosed by general practitioners. Selected cutaneous infections were cellulitis and varicella zoster (VZ). Non-cutaneous infections were gastroenteritis and urinary tract infection (UTI). A separate analysis was carried out for each outcome.

The morbidity codes used to identify infections were developed by those with clinical experience using a consensus procedure ^3-6^. For cellulitis and VZ we used the date of the first record. For gastroenteritis and UTI, we assumed infections separated by 28 days or less represented the same episode of infection, and defined the incident date as the first in the series of consecutive records separated by 28 days or less, using the first record of gastroenteritis and UTI during follow-up as the outcome (including individuals with episodes of infection prior to start of follow up, but excluding those with episodes of infection recorded on the day of the start of follow up, or within the 28 days before start of follow up).

Gastroenteritis was defined using a previously developed algorithm ^7^. The algorithm used morbidity codes recorded in primary care classified as: 1) definite codes (codes definitely representing gastrointestinal infection [e.g., J43..1, Gastroenteritis]); 2) symptom (codes representing symptoms of gastroenteritis [e.g., 1992 – vomiting]); and 3) pathogens (codes representing specific pathogens that can cause gastroenteritis [e.g., A3B4.00, Escherichia coli infection]). Gastroenteritis was defined as either: 1) a single definite gastroenteritis code recorded as part of a GP consultation; or 2) a combination of a symptom code followed by a pathogen code in the subsequent 28 days. Symptom codes must have been recorded as part of a GP consultation (i.e., not a letter).

Diagnostic code lists can be found at Morbidity code lists for all exposures, outcomes and covariates can be found at: <https://github.com/hendersonad/2021_SkinEpiExtract/tree/main/codelists>.

## Covariates

To avoid collider bias we selected covariables guided by a literature review and construction of a theoretical framework (Supplementary Methods Figure 2).

**Supplementary Methods Figure 2: Causal Diagram**

Theoretical framework showing the hypothesized relationships between atopic eczema (exposure), potential confounders, effect modifiers, mediators, and infection (outcome [cellulitis, varicella zoster, gastroenteritis, urinary tract infection])


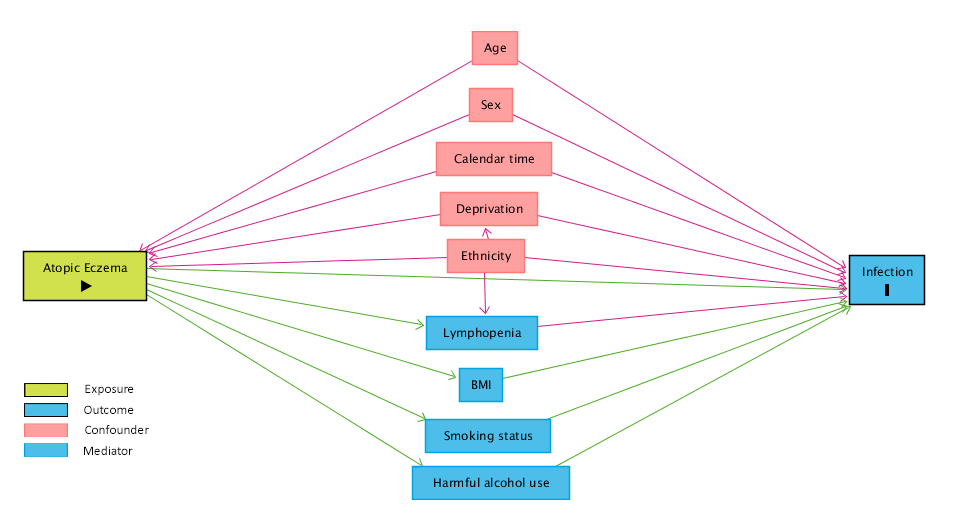


NB: Figure only includes variables that could be robustly captured using routinely collected primary care data. The theoretical framework is a simplified visualisation of the relationship between exposure, outcome and potential confounders and mediators. Variables included may have a more complicated interrelationship with the covariates.

Age, calendar period, sex, level of deprivation (as quintiles of Carstairs index) and ethnicity were deemed to be plausibly associated with both exposure (AE) and outcome (infection) and not on the causal pathway (i.e., potential confounders). We considered lymphopenia, body mass index (BMI), smoking status and harmful alcohol use as possible mediators of the association between AE and infection.

Read codes for BMI are rarely recorded and not used. BMI was calculated using height and weight measures recoded closest to cohort entry date (in order of preference records: from 1 year before or 1 month after cohort entry were the first choice; followed by records from 1 month to 1 year from cohort entry; then any records prior to the year before cohort entry date; and finally records more than 1 year after cohort entry date) ^8, 9^. BMI categories were divided according to the World Health Organization classification ^10^: underweight (<18.5kg/m^2^), normal weight (18.5-24.9kg/m^2^), overweight (25-29.9kg/m^2^) and obese (>30kg/m^2^). We only used records for BMI of between 10kg/m^2^ and 80kg/m^2^ as we regarded anything below 10kg/m^2^ or above 80kg/m^2^ to be implausible.

We defined smoking status as non-smokers, and ex or current smokers. We identified harmful alcohol use using Read codes suggesting harmful or heavy alcohol use, or a prescription for drugs used to maintain abstinence (e.g., acamprosate). This was a time-updated variable and individuals were considered to have harmful alcohol use after date of diagnosis.

## Lymphopenia

We used blood test results recorded in primary care records to identify individuals with lymphopenia. Individuals were considered to have lymphopenia if a lymphocyte count was less than 1 x 10^9^/L. Time with lymphopenia was defined as 6 months (182.63 days) after a test result indicating lymphopenia. If second blood test demonstrating lymphopenia was recorded within the 6 months following initial a lymphopenia result, participants were continued to be regarded as having lymphopenia for a further 6 months from the time of the second blood test (and so on if there were subsequent lymphopenia results within another 6 months).

## Statistical Analysis

Before the start of the study, we calculated that the minimum detectable HR for associations with infections ranged from 1.21 for common infections (ie. 1000 per 100,000 personyears) to 2.45 for less common infections (i.e. 40 per 100,000 personyears).

We assessed the effect of AE exposure on each outcome (cellulitis, VZ, gastroenteritis or UTI) using Cox regression stratified by matched set to obtain the hazard ratios (HZ) and 95% confidence interval (95%CI). Analyses implicitly adjusted for matching variables (sex, general practice), and age (underlying timescale). In sequential models, we additionally adjusted for: 1) potential confounders (deprivation and calendar time); 2) potential mediators of the relationship between AE and specific infections (BMI, smoking and harmful alcohol use); and 3) finally, we also further adjusted for time with lymphopenia. To preserve matching, analyses only included valid matched sets; that is, entire matched sets were excluded if the AE-exposed individuals were excluded, or if no individuals without AE remained in the set.

We calculated the absolute incidence rate of each infection directly among those with AE. Meaningful infection incidence rates in people without AE could not be reliably estimated due to matching (individuals without AE were not representative of the general population). Therefore, we estimated the incidence rate in those without AE by multiplying the rates in those with AE by the corresponding estimated HR of the confounder-adjusted model (after inverting it to compare unexposed with exposed) ^9, 11^. We then calculated the attributable risk as the difference between the incidence rate in individuals with and without AE. The population-attributable risk fraction (PAF) was estimated using the HR of the confounder-adjusted model and under several assumptions (i.e., the relationship was causal, AE and infections were recoded accurately, infections in individuals with AE can be prevented by eliminating AE, and that the prevalence of AE in the population was10%) ^12, 13^.

## Sensitivity analyses

We conducted a series of sensitivity analyses to explore possible sources of bias introduced including: 1) limiting to individuals with no previous diagnosis of lymphoma; 2) only including those entering the cohort after 2006 when ethnicity was more repletely recorded and adjusting for ethnicity; and 3) additionally adjusting for high-dose corticosteroid use in the models with mediators and time with lymphopenia.

## Secondary analyses

In secondary analyses, we: 1) redefined AE exposure using AE severity as a time-updated variable and compared incidence rates of specific infections in those with mild, moderate, or severe AE to those with no AE; and 2) explored (in separate analyses) whether the relationship between AE and specific infection was modified by sex, age and asthma status.

To assess whether increasing AE severity was associated with different rates of infection, we defined eczema as being mild, moderate, or severe AE. Individuals meeting neither moderate nor severe definitions were classified as having mild AE.

Individuals was classified as having moderate AE at the first of: 1) Second potent topical corticosteroid prescription within one year, or 2) first topical calcineurin inhibitor prescription. Individuals was classified as having severe AE at the first of: 1) first systemic treatment, excluding oral corticosteroid, 2) first phototherapy code, or 3) first referral for AE to secondary care ^9, 11, 14, 15^.

All p-values were based on likelihood-ratio tests with 95%CI. Statistical analyses were performed using STATA 16 (StataCorp LLC, College Station, TX, USA).

## Ethical approval

Ethical approval was obtained from the Independent Scientific Advisory Committee (ISAC), which oversees the research CPRD data (Protocol number: 18_104), and LSHTM ethics committee (Reference: 25683).

# **Replication using data from the** **National Health and Nutrition Examination Survey (NHANES)**

We sought to validate our primary findings in another population-based setting. Akin to the primary analysis, the aim of this validation analysis was to determine if eczema is associated with lymphopenia.

## Setting and study population

Publicly available data from the US-based National Health and Nutrition Examination Survey (NHANES) were pooled for the survey years 1999-2006. The original cohort protocol was approved by the National Center for Health Services Research Ethics Review Board. Briefly, NHANES is a series of cross-sectional surveys conducted by the National Center for Health Statistics (NCHS) through household interviews, blood sampling, and physical examinations. NHANES uses a multistage probability design to select a nationally-representative sample of the non-institutionalized, civilian US population. Details on 1999-2006 NHANES surveys design and methodology are presented elsewhere^16^. We included all participants who were 18 years and older.

## Eczema

Eczema questions were assessed using interview questionnaires. Eczema was defined in the following three ways for survey years 2005-2006: 1) Doctor-diagnosed ever having eczema using the question, “Has a doctor or other health professional ever told you that you have eczema?”; 2) Eczema in the past year was assessed using the questions, “Have you ever had an itchy rash which was coming and going for at least 6 months?” and “Have you had this itchy rash at any time in the last 12 months?”; and 3) Flexural eczema was assessed with the addition of the question, “Has this itchy rash at any time affected any of the following places: the folds of the elbows, behind the knees, in front of the ankles, under the buttocks, or around the neck, ears, or eyes?” For the survey years 1999-2004, eczema in the past year was assessed with the question, “During the past 12 months, have you had dermatitis, eczema, or any other type of red, inflamed skin rash?” Because the questions were different for years 2005-2006 and 1999-2004, we conducted separated analyses for 2005-2006 and a pooled analyses for 1999-2006 using eczema in the past year.

## Blood cell counts

Blood specimens were collected during Mobile Examination Center (MEC) examinations, which is conducted around the same time as the interviews. MECS are traveling clinics used by the NCHS to collect participant health and laboratory data. The Beckman Coulter MAXM instrument was used to produce a completed blood count and provide blood cell distributions. Absolute lymphocyte count (1,000 cells/uL) was calculated using the following formula: White blood cell count (1,000 cells/uL) * Lymphocyte percent (%)/100. Lymphopenia was defined as having fewer than 1,000 lymphocytes per microliter^17^. We excluded participants whose absolute lymphocyte count was above 4,800 lymphocytes per microliter, indicating a presence of infection.

## Covariates

We adjusted for the same covariates used in the main analysis: age, sex, and smoking. For the analysis with lymphopenia as the outcome, we additionally adjusted for glucocorticoid use. In the pooled analysis including data from 1999-2006, we also additionally adjusted for wave of data collection. We used logistic regression to estimate the association between eczema and lymphopenia, adjusting for covariates. Multivariable linear regressions were used when the outcome was absolute lymphocyte count. All models used sampling weights developed for NHANES to account for the complex survey design and oversampling of certain participant groups. Participants who had missing data on lymphocyte counts, eczema, or covariates were excluded.

All statistical analyses were conducted using Stata, version16. Statistical significance was set to be P-values <0.05.

# **References**

1. Gelfand JM, Troxel AB, Lewis JD, Kurd SK, Shin DB, Wang X, et al. The risk of mortality in patients with psoriasis: results from a population-based study. Arch Dermatol 2007; 143:1493-9.

2. Abuabara K, Magyari AM, Hoffstad O, Jabbar-Lopez ZK, Smeeth L, Williams HC, et al. Development and Validation of an Algorithm to Accurately Identify Atopic Eczema Patients in Primary Care Electronic Health Records from the UK. J Invest Dermatol 2017; 137:1655-62.

3. Forbes H. Clinical Code List - Acute Gastroenteritis - Read Codes. London, United Kingdom: London School of Hygiene & Tropical Medicine, 2019.

4. Crellin E, Mansfield KE, Leyrat C, Nitsch D, Douglas IJ, Root A, et al. Clinical Code List - Read Codes - Urinary Tract Infection. London, United Kingdom: London School of Hygiene & Tropical Medicine, 2017.

5. Forbes H. Clinical Code List - Zoster - Read Codes. London, United Kingdom: London School of Hygiene & Tropical Medicine, 2019.

6. Muzambi R, Bhaskaran K, Smeeth L, Warren-Gash C. Clinical codelist - Skin and soft tissue infections read codes. London, United Kingdom: London School of Hygiene & Tropical Medicine, 2020.

7. Mansfield KE, Douglas IJ, Nitsch D, Thomas SL, Smeeth L, Tomlinson LA. Acute kidney injury and infections in patients taking antihypertensive drugs: a self-controlled case series analysis. Clin Epidemiol 2018; 10:187-202.

8. Bhaskaran K, Douglas I, Forbes H, dos-Santos-Silva I, Leon DA, Smeeth L. Body-mass index and risk of 22 specific cancers: a population-based cohort study of 5&#xb7;24 million UK adults. The Lancet 2014; 384:755-65.

9. Schonmann Y, Mansfield KE, Hayes JF, Abuabara K, Roberts A, Smeeth L, et al. Atopic Eczema in Adulthood and Risk of Depression and Anxiety: A Population-Based Cohort Study. The journal of allergy and clinical immunology. In practice 2020; 8:248-57.e16.

10. Nutrition - Body Mass Index.] Available from <https://www.euro.who.int/en/health-topics/disease-prevention/nutrition/a-healthy-lifestyle/body-mass-index-bmi>.

11. Silverwood RJ, Forbes HJ, Abuabara K, Ascott A, Schmidt M, Schmidt SAJ, et al. Severe and predominantly active atopic eczema in adulthood and long term risk of cardiovascular disease: population based cohort study. BMJ 2018; 361:k1786.

12. Silverberg JI. Public Health Burden and Epidemiology of Atopic Dermatitis. Dermatol Clin 2017; 35:283-9.

13. Silverberg JI, Hanifin JM. Adult eczema prevalence and associations with asthma and other health and demographic factors: a US population-based study. The Journal of allergy and clinical immunology 2013; 132:1132-8.

14. Ascott A, Mansfield KE, Schonmann Y, Mulick A, Abuabara K, Roberts A, et al. Atopic eczema and obesity: a population-based study. Br J Dermatol 2021; 184:871-9.

15. Mansfield KE, Schmidt SAJ, Darvalics B, Mulick A, Abuabara K, Wong AYS, et al. Association Between Atopic Eczema and Cancer in England and Denmark. JAMA Dermatology 2020; 156:1086-97.

16. Zipf G, Chiappa M, Porter KS, Ostchega Y, Lewis BG, Dostal J. National health and nutrition examination survey: plan and operations, 1999-2010. Vital Health Stat 1 2013:1-37.

17. .] Available from <https://www.nhlbi.nih.gov/health-topics/lymphocytopenia>.
