# Supplementary material for: The association between atopic eczema and lymphopenia: Results from a UK cohort study with replication in US survey data
Source: J Eur Acad Dermatol Venereol. 2023 Jan 25;37(6):1190–8. doi: 10.1111/jdv.18841 (PMC10947025; doi:10.1111/jdv.18841)
Supplement: Supplementary file 14 — Appendix S2 [file JDV-37-1190-s007.docx]

Supplementary Results

# **Results for secondary analysis focused on infection risk**

To assess gastroenteritis and UTI outcomes we identified cohorts including 1,034,905 people with AE and 4,999,500 without. After excluding people with previous cellulitis, we were left with 934,417 people with AE and 4,312,661 without for analyses with cellulitis as the outcome. Finally, after excluding individuals with a history of VZ, we identified 960,516 individuals with AE and 4,461,484 without for analysis of VZ as the outcome (Supplementary Results Figure 1).

**Supplementary Results Figure 1: Flowchart**

Detailed flow diagram of the creation of the cellulitis, varicella zoster, gastroenteritis and urinary trac infection cohort, and reasons for exclusion

**
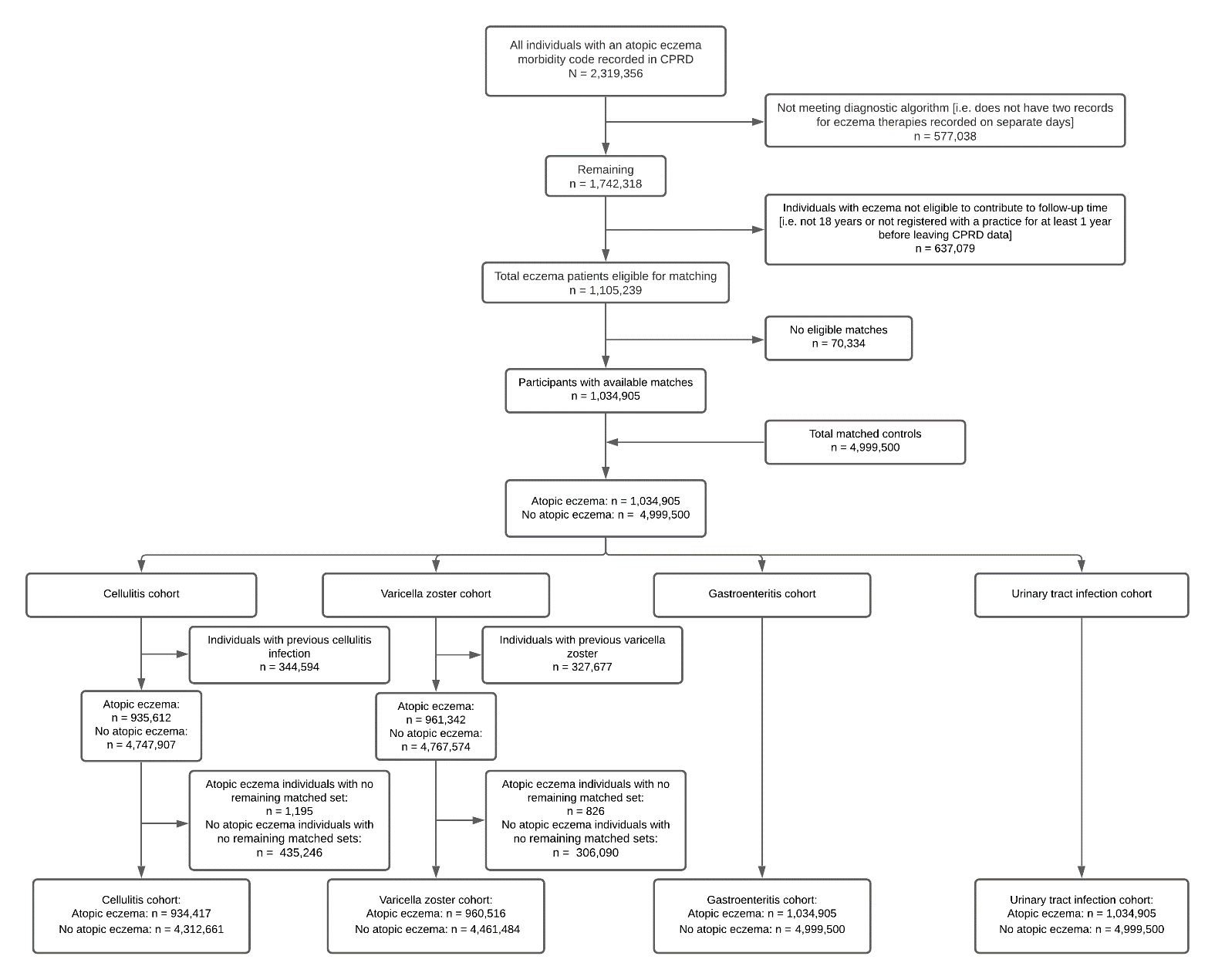
**

Median follow-up was shorter in people without AE (between 4 (IQR: 2-9) and 5 (IQR: 2-10) years) than people with AE (between 4 (IQR: 2-9) and 6 (IQR: 2-10 years). People with and without eczema contributed a similar distribution of person time across age bands and calendar periods. Individuals with and without AE were predominantly women (approximately 58%).

People AE were more likely to be classified as ex or current smokers (individuals in cohorts assessing cellulitis: 45.03% vs 42.37%, VZ: 46.19% vs 43.71%, gastroenteritis: 45.47% vs 42.90%, and UTI: 45.47% vs 42.90%) and were more likely to have a diagnosis of asthma (individuals in cohorts assessing cellulitis: 25.81% vs 13.53%, VZ: 25.16% vs 13.32%, gastroenteritis: 25.77% vs 13.65%, and UTI: 25.55% vs 13.51%) or harmful alcohol use (cellulitis: 6.89% vs 5.67%, VZ: 7.36% vs 6.07%, gastroenteritis: 7.05% vs 5.81%, and UTI: 6.86% vs 5.60%) compared to people without AE. BMI distribution was similar for people with and without AE . The median time with lymphopenia across all cohorts were higher in people with AE (between 108 to 112 days) compared to those without (between 104 to 108 days).

## Main analysis

The HR (95%) comparing rate of common infections in individuals with AE compared to those without after implicitly adjusting for age, sex, date of cohort and GP practice and explicitly adjusting for calendar period and Carstairs Index was: cellulitis 1.64 (95% CI 1.62-1.66) , VZ 1.16 (95% CI 1.11-1.21) , gastroenteritis 1.38 (95% CI 1.36-1.39), and UTI 1.22 (95% CI 1.22-1.23) (Supplementary Results Figure 2).

**Supplementary Results Figure 2**: **Association between AE and infections**

Forest plot of the hazard ratios (95%CI) for rates of specific infections in people with atopic eczema compared to those without.


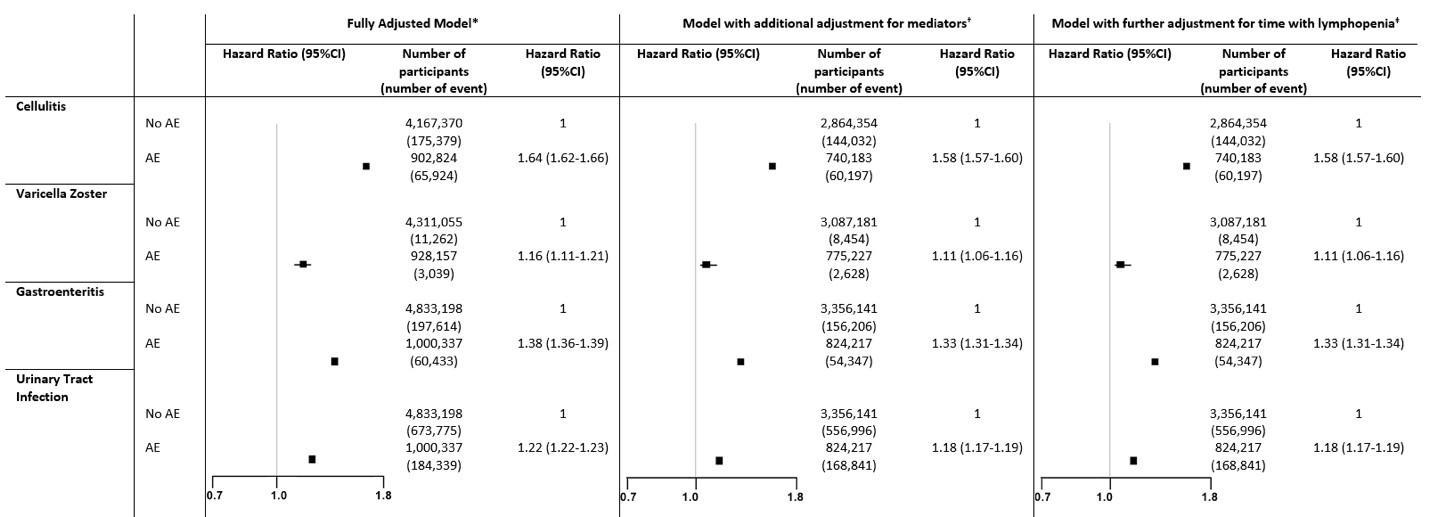


AE, atopic eczema

All models were fitted to participants with complete data for all included variables. Only sets with at least one exposed and unexposed individual were included. Hazard ratios were calculated from a Cox regression mode with age as the underlying timescale and stratified by matching factors (age, sex, date of entry into cohort, and general practice).

^*^ Model implicitly adjusting for matching factors and explicitly adjusting for calendar period and Carstairs index

^†^ The model adds smoking status, body mass index and time-updated harmful alcohol use.

^‡^ The model with the addition of time with lymphopenia

All p-values were <0.0001 unless stated otherwise (likelihood ratio test comparing the model with exposure variable to a model without the exposure variable)

HRs for all four infections were attenuated after additionally adjusting for lifestyle factor mediators (BMI, smoking status, and harmful alcohol use) (cellulitis: HR, 1.58; 95% CI 1.57-1.60; VZ: 1.11; 95% CI 1.06-1.16; gastroenteritis: HR, 1.33; 95% CI 1.31-1.34; and UTI: HR, 1.18; 95% CI 1.17-1.19). HR estimates for all four infections were unchanged after further adjusting for time with lymphopenia.

The absolute excess rate of infection that could be due to AE (attributable risk) was: cellulitis 44.10 per 10,000 person-years at risk (PYAR) (95%CI 43.25 -44.93), VZ 0.65 per 10,000 PYAR (95%CI 0.47-0.82), gastroenteritis 25.39 per 10,000 PYAR (95%CI 24.41-25.87), and UTI 54.73 per 10,000 PYAR (95%CI 54.73-56.75).

Sensitivity analyses showed broadly similar effect estimates to those from the main analysis.

## Secondary analyses

#### Atopic eczema severity

For cellulitis, gastroenteritis and UTI, regardless of AE severity level, we saw evidence of an association between AE and infection. However, for VZ, we saw evidence of association between AE and VZ after adjusting for confounders, but there was no evidence of an association after further adjusting for mediators and time with lymphopenia (Supplementary Results Figure 3).

**Supplementary Results Figure 3:** **Associations between AE and infections stratified on AE severity**

A Forest plot showing the association between time-updated atopic eczema severity and specific infections.


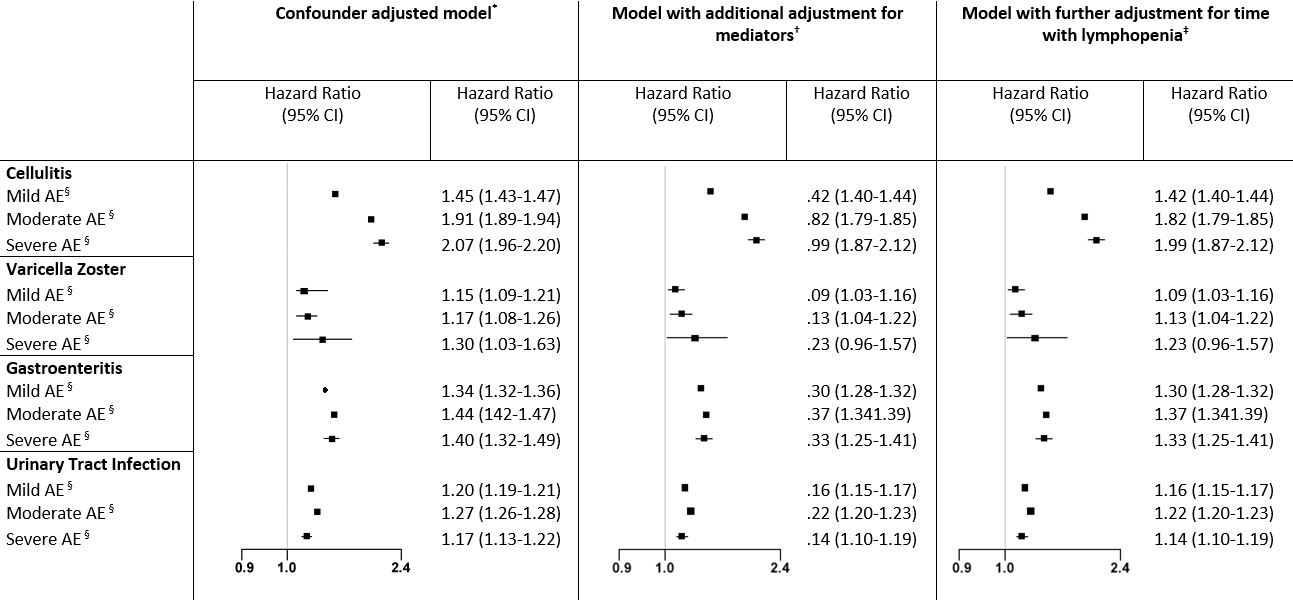


AE, atopic eczema

All models were fitted to participants with complete data for all included variables. Only sets with at least one exposed and unexposed individual were included. Hazard ratios were calculated from a Cox regression mode with age as the underlying timescale and stratified by matching factors (age, sex, date of entry into cohort, and general practice).

^*^ Model implicitly adjusting for matching factors and explicitly adjusting for Carstairs Index and calendar period.

^†^ The model adds lifestyle factors.

^‡^ The model with the addition of time with lymphopenia.

^§^ vs no atopic eczema

All p-values were <0.0001 unless stated otherwise (likelihood ratio test comparing the model with severity variable to a model without the severity variable)

p-values for the adjusted model adjusting for potential mediators and the model further adjusting for lymphopenia were 0.0002 for varicella zoster (likelihood ratio test comparing model with severity variable to a model without the severity model)

The association between AE and cutaneous infections increased with increasing AE severity. For example, the HRs for cellulitis in people with eczema compared to people without were: 1.45 (95%CI 1.43-1.47) in people with mild AE, 1.91 (95%CI 1.89-1.94) in people with moderate AE, and 2.06 (95%CI 1.96-2.20) in people with severe AE. Compared to people without AE, the HRs for non-cutaneous infections (gastroenteritis and UTI) were highest in individuals with moderate AE.
